# Structure basis for the modulation of CXC chemokine receptor 3 by antagonist AMG487

**DOI:** 10.1038/s41421-023-00617-0

**Published:** 2023-11-28

**Authors:** Haizhan Jiao, Bin Pang, Ying-Chih Chiang, Qiang Chen, Qi Pan, Ruobing Ren, Hongli Hu

**Affiliations:** 1 Kobilka Institute of Innovative Drug Discovery, School of Medicine, The Chinese University of Hong Kong, Shenzhen, Shenzhen, Guangdong China; 2 School of Life and Health Sciences, School of Medicine, The Chinese University of Hong Kong, Shenzhen, Shenzhen, Guangdong China; 3 Shanghai Key Laboratory of Metabolic Remodeling and Health, Institute of Metabolism and Integrative Biology, Fudan University, Shanghai, China; 4 Shanghai Qi Zhi Institute, Shanghai, China

**Keywords:** Cryoelectron microscopy, Immunology, Cell signalling

Dear Editor,

CXC chemokine receptor 3 (CXCR3) and three interferon-induced CXC chemokines, specifically CXCL9 (Mig), CXCL10 (IP-10), and CXCL11 (I-TAC), are strongly associated with the migration of CD4^+^ Th1 cells and CD8^+^ cytotoxic T lymphocytes in immune responses^1,2^. The physiological and pathological functions of CXCR3 have been studied in infection, cancer, autoimmune diseases, and transplant rejection^2–5^. Only one CXCR3 antagonist, AMG487, has been evaluated in clinical trials for psoriasis and rheumatoid arthritis. AMG487 is a quinazolinone derivative that could prevent the binding of CXCL10 and CXCL11 to CXCR3 with high selectivity^6^. To date, the mechanism of the antagonism of AMG487 remains unclear. Here we determined the structure of CXCR3 complexed with AMG487 and the structure of the CXCR3–DNG_i_ complex activated by CXCL10. The molecular mechanism of CXCR3 inhibition by AMG487 is elucidated, and we believe that our study will provide insightful perspectives for developing CXCR3-targeting antagonists.

The method of coupling a nanobody Nb6 to the receptor was used to facilitate the cryo-EM analysis. The ICL3 of CXCR3 was replaced by the ICL3 of the kappa opioid receptor. The chimeric receptor retained comparable response to CXCL10 (Supplementary Fig. S1). CXCR3^κOR^ was purified in the presence of AMG487 and coupled to nanobody Nb6 (Supplementary Fig. S2a, b). Single particle analysis of the purified complex yields a density map at 3.0 Å resolution (Supplementary Fig. S2c–g and Supplementary Table S1). In the density map, the transmembrane helices could be distinguished, and residues 54–338 could be traced (Fig. 1a, b and Supplementary Fig. S2h). A density in the central pocket was found to be suitable for accommodating AMG487 (Fig. 1b).

**Fig. 1:**
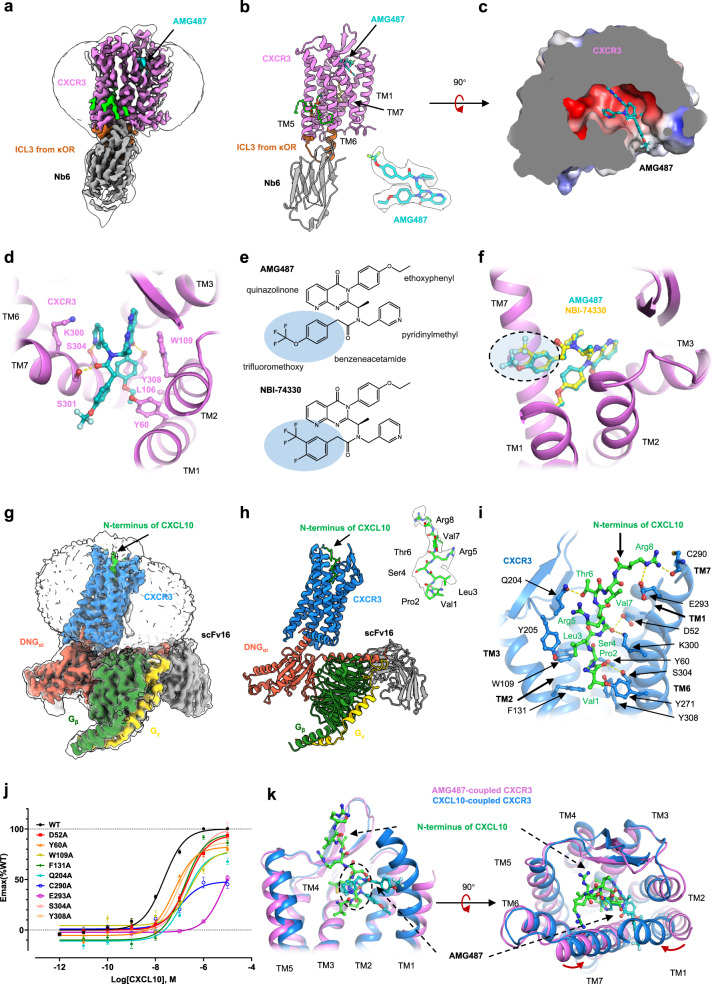
The molecular basis for the antagonism of AMG487. **a** The density map of CXCR3^κOR^–AMG487–Nb6. **b** The atomic model of CXCR3^κOR^–AMG487–Nb6. CXCR3, ICL3 from κOR, and Nb6 are shown as cartoons. AMG487, cholesterol, phosphatidylcholine, and lysophosphatidylcholine are shown as sticks. The density of AMG487 is presented. In **a** and **b**, CXCR3, ICL3 from κOR, Nb6, AMG487, cholesterol, phosphatidylcholine, and lysophosphatidylcholine are colored violet, brown, gray, cyan, yellow, green, and dark green, respectively. **c** The surface electronic potential of CXCR3 near the AMG487 binding pocket. AMG487 is shown as sticks and colored cyan. **d** Interactions between CXCR3 and AMG487. Residues involved in ligand binding are shown as sticks, and interactions are indicated by yellow dashes. **e** The chemical structures of AMG487 and NBI-74330. **f** The potential binding pattern of NBI-74330. CXCR3 is shown as cartoon and colored violet. AMG487 and NBI-74330 are shown as sticks and colored cyan and yellow, respectively. **g** Density map of the CXCR3–CXCL10–DNG_i_–scFv16 complex. **h** Cartoon model of the CXCR3–CXCL10–DNG_i_–scFv16 complex. The density of the N-terminus of CXCL10 is presented. In **g** and **h**, CXCR3, DNGα_i_, G_β_, G_γ_, scFv16, and CXCL10 are colored blue, red, dark green, yellow, gray, and green, respectively. **i** Interactions between CXCR3 and the N-terminus of CXCL10. CXCR3 is shown as cartoon and colored blue, and the N-terminus of CXCL10 is shown as sticks and colored green. Residues involved in interactions are shown as sticks, and interactions are indicated by yellow dashes. **j** cAMP responses of CXCR3 mutants to CXCL10. cAMP responses are normalized to the percent agonist activity of wild-type CXCR3. Data were presented as mean ± s.e.m. (*n* = 6). **k** Comparison of the overall structure of active (blue) and inactive (violet) CXCR3. AMG487 (cyan) and the N-terminus of CXCL10 (green) are shown as sticks. The overlap of AMG487 and CXCL10 is indicated by a circle.

In the CXCR3^κOR^–AMG487–Nb6 complex, AMG487 is trapped in a negatively charged pocket in CXCR3, with a buried surface area of 481.6 Å^2^ (Fig. 1c). The binding pocket is open to the lipid bilayer through a cleft between TM1 and TM7. The trifluoromethoxy (OCF3) group is buried in the cleft and faces toward the lipid bilayer (Fig. 1c, d). AMG487 is sandwiched between TM7 on one side and TM1 and TM2 on the other side (Fig. 1d). The aza-quinazolinone group is mainly stabilized by stacking with Trp109^2.60^ and is hydrogen bonded to the side chain of Tyr308^7.43^ (Fig. 1d and Supplementary Fig. S3a). The ethoxyphenyl group is stabilized by hydrophobic interactions with Tyr60^1.39^, Leu106^2.57^, and Trp109^2.60^, while the benzene ring of the ethoxyphenyl group stacks with the benzene ring of Tyr308^7.43^ (Fig. 1d and Supplementary Fig. S3a). The pyridinylmethyl group is sandwiched between the side chains of Lys300^7.35^ and Ser304^7.39^ in TM7 and forms a hydrogen bond with the side chain of Ser304^7.39^ (Fig. 1d and Supplementary Fig. S3a). The hydroxy group of Tyr60^1.39^ points towards the center of the benzene ring in the benzeneacetamide group, and the carbonyl group is hydrogen bonded to the side chain of Ser301^7.36^ (Fig. 1d and Supplementary Fig. S3a).

NBI-74330 is a CXCR3 antagonist that shows therapeutic potential in animal models of atherosclerosis and arthritis^7,8^. In AMG487, the 4’ position of the benzene ring is occupied by an OCF3 group (Fig. 1e). In NBI-74330, a fluorine atom occupies the 4’ position and a trifluoromethyl (CF3) group occupies the 3’ position of the benzene ring (Fig. 1e). When docking NBI-74330 into the density of AMG487 in Coot, the posture was very similar to that of AMG487 (Fig. 1f). Therefore, we believe that NBI-74330 may inhibit CXCR3 in a way similar to AMG487. By comparing the structures of chemokine receptors complexed with antagonists, we found that the binding pocket of AMG487 in CXCR3 is largely overlapped with that of MK-0812 and BMS-681 in CCR2 (Supplementary Fig. S3b)^9,10^. In comparison, the binding pocket of AMG487 only partially overlaps with that of the CCR5 antagonists maraviroc, compound 21, and compound 34, and the CXCR4 antagonist IT1t (Supplementary Fig. S3c, d). The CF3 groups in AMG487, MK-0812, and BMS-681 occupy similar positions between TM1 and TM7 (Supplementary Fig. S3b). Residues 1.39, 2.60, 7.36, 7.39, and 7.43 involved in interactions with AMG487 are also in contact with MK-0812 and BMS-681 (Supplementary Fig. S3e, f). Among these residues, 1.39 and 2.60 are identical in CXCR3 and CCR2, and residues 7.36, 7.39, and 7.43 on TM7 are Ser301, Ser304, and Tyr308 in CXCR3, but Gln288, Glu291, and Met295 in CCR2 (Fig. 1d and Supplementary Fig. S3e, f). The comparison suggests that AMG487 adopts an antagonistic mechanism similar to MK-0812 and BMS-681 but possesses some distinct features.

The CXCR3–CXCL10–DNG_i_ complex was stabilized by the NanoBit tethering strategy. The complex was purified and further stabilized by scFv16 (Supplementary Fig. S4a, b). A density map at 3.2 Å resolution was obtained through single particle analysis (Supplementary Fig. S4c–g and Supplementary Table S1). The densities of the transmembrane helices could be distinguished, and the atom model was built accordingly (Fig. 1g, h and Supplementary Fig. S4h). For CXCL10, however, only eight residues in the proximal N-terminus could be recognized (Fig. 1h), suggesting that CXCL10 binds to CXCR3 with high flexibility.

The N-terminus of CXCL10 is surrounded by TM1, TM2, TM3, TM6, and TM7 (Fig. 1i). An uncharged “VPLS” motif and a positively charged “RTVR” motif could be distinguished. In the “VPLS” motif, Val1^CXCL10^ and Pro2^CXCL10^ insert most deeply and form hydrophobic interactions with Tyr60^1.39^, Trp109^2.60^, Phe131^3.32^, Tyr271^6.51^, and Tyr308^7.43^ (Fig. 1i). The main chain carbonyl group of Val1^CXCL10^ is hydrogen bonded to the hydroxyl group of Ser304^7.39^ (Fig. 1i). Tyr205^ECL2^ is involved in hydrophobic stacking with Leu3^CXCL10^, while Ser4^CXCL10^ is coordinated by Asp52^1.31^ and Lys300^7.35^ (Fig. 1i). In the “RTVR” motif, the hydroxyl group of Thr6^CXCL10^ is hydrogen bonded to the side chain of Gln204^ECL2^ (Fig. 1i). Arg8^CXCL10^ is in contact with the side chain of Glu293^7.28^ and the main chain of Cys290^7.25^ (Fig. 1i). Mutation of Asp52^1.31^, Tyr60^1.39^, Trp109^2.60^, Phe131^3.32^, Gln204^ECL2^, Cys290^7.25^, Glu293^7.28^, Ser304^7.39^, and Tyr308^7.43^ resulted in decreased potency of CXCL10 (Fig. 1j and Supplementary Table S2), suggesting these residues are crucial for CXCL10 binding and receptor activation. Truncation of two amino acids in the N-terminus of CXCL10 resulted in reduced CXCR3-binding properties, loss of calcium signaling capacity, and a 30-fold reduction in chemotactic activity^11^. Mutation of Arg5 and Arg8 in the N-terminus of CXCL10 resulted in a 7- and 60-fold increase in IC_50_^12^. Therefore, both the uncharged VPLS motif and the positively charged RTVR motif in the N-terminus of CXCL10 are critical for the ligand binding and receptor activation. Due to the sequence similarity of the N-terminus of CXCL9, CXCL10, and CXCL11 (Supplementary Fig. S5a), the structure of the CXCR3–CXCL10–DNG_i_ complex may provide insight into the binding pattern of the N-terminus of CXCL9 and CXCL11.

Compared to AMG487-coupled CXCR3, the extracellular region of CXCL10-coupled CXCR3 is more compact with the inward movement of TM2 and TM5 (Supplementary Fig. S5b). On the intracellular side, the outward wing of TM6 releases the packing between TM3 and TM6, exposing the binding cavity for the G_αi_ protein (Supplementary Fig. S5c, d). The conformational change of TM6 is accompanied by the inward displacement of TM3, TM5, and TM7 (Supplementary Fig. S5c, d). MD simulations indicate that the apo CXCR3 generally undergoes greater movement than the AMG487-bound CXCR3 (Supplementary Fig. S5e–g). In a snapshot of the apo CXCR3 simulation (Supplementary Fig. S5h), movements on the extracellular side of TM1, TM6, and TM7, and small shifts on the intracellular side of TM7 are observed. Therefore, we suggest that the binding of AMG487 may stabilize the inactive conformation. In addition, the binding pockets of AMG487 and CXCL10 partially overlap (Fig. 1k). Several residues (Tyr60^1.39^, Trp109^2.60^, Ser304^7.39^, and Tyr308^7.43^) that are critical for chemokine binding and the activation of CXCR3 are occupied by AMG487 in the inactive state. The observation is consistent with the previous study that AMG487 could compete with CXCL10 by binding to the orthosteric pocket^6^.

According to the mass spectrum analysis (Supplementary Fig. S6a, b) and electron densities (Supplementary Fig. S6c–e), several lipids were identified in the density map of the CXCR3^κOR^–AMG487–Nb6 complex. Firstly, a cholesterol molecule is trapped in the cavity surrounded by TM2, TM3, and TM4 (Supplementary Fig. S6c). Allosteric antagonists AZ3451 and ORG27569 were found to bind to PAR2 and CB1 receptors in similar sites (Supplementary Fig. S6c). Secondly, in the cavity defined by TM3, TM4, and TM5, a lysophosphatidylcholine molecule is found (Supplementary Fig. S6d). This cavity is also well-known for allosteric antagonist development, such as NDT9513727/avacopan targeting C5aR and AS408 targeting β_2_-AR (Supplementary Fig. S6d). In addition, a phosphatidylcholine binding site surrounded by TM3, TM5, and TM6 was identified (Supplementary Fig. S6e). The entrance to the phosphatidylcholine binding pocket between TM5 and TM6 is guarded by Leu228^5.51^ and Ala265^6.45^. Compared to the inactive structure of CXCR2 and CXCR4^13,14^, the distances between the α-carbon atoms of 5.51 and 6.45 are comparable (Supplementary Fig. S6f–h). However, the side chains of 5.51 and 6.45 in CXCR3 are smaller and the distance between the side chains is obviously larger. Therefore, we suggest that the smaller residues in CXCR3 make the cavity able to accommodate the lipids. This lipid-binding site has not been characterized in other GPCRs. Further studies are required to verify the binding and functions of these lipids in the wild-type receptor and to explore the druggability of these lipid-binding sites.

### Supplementary information

Supplementary figures and tables

## Data Availability

The coordinates and maps for the CXCR3^κOR^–AMG487–Nb6 and CXCR3–CXCL10–DNG_i_–scFv16 complex structure have been deposited in the Protein Data Bank/Electron Microscopy Data Bank under accession code 8K2W/36841 and 8K2X/36842, respectively.
