# Supplementary material for: Structure basis for the modulation of CXC chemokine receptor 3 by antagonist AMG487
Source: Cell Discov. 2023 Nov 28;9:119. doi: 10.1038/s41421-023-00617-0 (PMC10682378; doi:10.1038/s41421-023-00617-0)
Supplement: Supplementary file 1 — Supplementary figures and tables [file 41421_2023_617_MOESM1_ESM.pdf]

Methods

Expression and purification of CXCR3$^{\kappa OR}$-AMG487-Nb6 complex

Wild-type human CXCR3 (residues 1-361) was cloned into pFastBac 1 vector with a HA signal peptide followed by a Flag tag. The ICL3 of CXCR3 (residue 241-254) was replaced with the ICL3 from the kappa-opioid receptor (residue 252-273) to facilitate the cryo-EM analysis through the coupling of a nanobody named Nb6[1]. BRIL was fused to the N-terminus of the chimeric CXCR3$^{\kappa OR}$ to enhance the expression level. Nanobody Nb6 with an N-terminal GP67 signal peptide and a C-terminal 8×His tag was cloned into pFastBac 1 vector. CXCR3$^{\kappa OR}$ were expressed in Sf9 insect cells using the Bac-to-Bac baculovirus system, and cells were collected after incubation for 60 h at 27 °C. Nb6 was expressed in *Sf9* insect cells at 27 °C for 72 h.

The cell pellets expressing chimeric CXCR3$^{\kappa OR}$ were thawed in buffer containing 20 mM Hepes-Na, pH = 7.5, 300 mM NaCl, 1% LMNG, 0.2% CHS, 10% glycerol, and 4 μM AMG487 (MedChemExpress). After homogenization, the suspension was solubilized for 2 h at 4 °C and centrifuged at 40000 g for 30 min. The supernatant was collected and incubated with anti-DYKDDDDK G1 affinity resin (Genscript) for 1.5 h at 4 °C. The resin was washed in buffer 20 mM Hepes-Na, pH = 7.5, 100 mM NaCl, 0.1% LMNG, 0.02% CHS, 10% glycerol, and 4 μM AMG487, followed by buffer 20 mM Hepes-Na, pH = 7.5, 100 mM NaCl, 0.01% LMNG, 0.002% CHS, 10% glycerol, and 4 μM AMG487. Then the protein was eluted in buffer 20 mM Hepes-Na, pH = 7.5, 100 mM NaCl, 0.01% LMNG, 0.002% CHS, 10% glycerol, 4 μM AMG487, and 0.25 mg/mL DYKDDDDK peptide. The concentrated protein was incubated with Nb6 for 30 min on ice with a molar ratio of 1:1.5, and the CXCR3$^{\kappa OR}$-AMG487-Nb6 complex was further purified by size exclusion chromatography on Superose 6 10/300 Increase column in buffer 20 mM Hepes-Na, pH = 7.5, 100 mM NaCl, 0.005% LMNG, 0.001% CHS, and 4 μM AMG487. The peak fraction was collected and concentrated to around 5.0 mg/mL for cryo-EM sample preparation.

Cryo-EM sample preparation, data collection, and processing of the CXCR3$^{\kappa OR}$-AMG487-Nb6 complex

The CXCR3$^{\kappa OR}$-AMG487-Nb6 complex was applied to glow-discharged 300-mesh UltraAuFoil grids (UltraAuFoil-Au300-R1.2/1.3, Quantifoil) and subsequently vitrified using Vitrobot Mark IV (Thermo Fisher Scientific). The images were collected in the counted-Nanoprobe mode on a 300 kV

Titan Krios Gi3 electron microscope (Thermo Fisher Scientific) equipped with a Gatan K3 Summit detector and a GIF Quantum energy filter (Gatan). 7,223 movie stacks with 50 frames per movie were collected using SerialEM[2] software at super-resolution mode, with a pixel size of 0.425 Å and a defocus range of -1.5 µm to -2.0 µm. Each movie stack was recorded for 2.0 s with 0.04 s exposure per frame and a total dose of 51.21 $e^-/Å^2$.

Data processes were accomplished in RELION and CryoSPARC[3,4]. The beam-induced motion was corrected by MotionCor2[5] with the pixel size binned to 0.85 Å at the same time. Then the micrographs were imported into CryoSPARC, and contrast transfer function parameters were estimated by PatchCTF. 10,681,493 particles were auto-picked and extracted in a pixel size of 1.70 Å. After two rounds of 2D classification, 5,911,406 particles were selected. Four initial models were generated in CryoSPARC, and 834,686 particles were retained after excessive hetero-refinement in CryoSPARC. The particles were re-extracted in a pixel size of 0.85 Å. After non-uniform refinement and local refinement, a density map of 3.0 Å resolution was generated.

Expression and purification of CXCR3-CXCL10-DNG$_i$-scFv16 complex

Wild-type human CXCR3 (residues 1-361) was cloned into pFastBac 1 vector with a HA signal peptide followed by a Flag tag. The protein BRIL and LgBit were fused to the N-terminus and C-terminus of CXCR3, respectively. Domain negative $G_{\alpha i1}$ (DN$G_{\alpha i1}$) was cloned into the pFastBac 1 vector. $G_{\beta 1\gamma 2}$ with HiBit fusion to the C-terminus of $G_{\beta 1}$ was cloned into the pFastBac Dual vector. Human CXCL10 with a C-terminal 8×His tag, scFv16 with an N-terminal GP67 signal peptide and a C-terminal 8×His tag were cloned into pFastBac 1 vector, respectively. Bril-CXCR3-LgBit, DN$G_{\alpha i1}$, $G_{\beta 1\gamma 2}$-HiBit, and CXCL10 were co-expressed in *Sf9* insect cells at a virus ratio of 1:1:1:2. Cells were collected after incubation for 60 h at 27 °C. scFv16 was expressed in Sf9 insect cells at 27 °C for 72 h.

The cell pellets expressing CXCR3-CXCL10-DNG$_i$ were suspended and homogenized in buffer containing 20 mM Hepes-Na, pH = 7.5, 300 mM NaCl, 1% LMNG, 0.2% CHS, 10% glycerol, 5 mM $MgCl_2$, and 25 mU/mL apyrase. After solubilization for 2 h at 4 °C, the suspension was centrifugated at 40000 g for 30 min, and the supernatant was collected and incubated with anti-DYKDDDDK G1 affinity resin (Genscript) for 1.5 h at 4 °C. The resin was washed in buffer containing 20 mM Hepes-Na, pH = 7.5, 100 mM NaCl, 0.1% LMNG, 0.02% CHS, 10% glycerol, and 5 mM $MgCl_2$, followed by buffer containing 20 mM Hepes-Na, pH = 7.5, 100 mM NaCl, 0.01% LMNG, 0.002% CHS, 10%

glycerol, and 5 mM $MgCl_2$. Then the complex was eluted in buffer containing 20 mM Hepes-Na, pH = 7.5, 100 mM NaCl, 0.01% LMNG, 0.002% CHS, 10% glycerol, 5 mM $MgCl_2$, and 0.25 mg/mL DYKDDDDK peptide. The protein was concentrated and incubated with scFv16 for 30 min on ice with a molar ratio of 1:1.5. The complex was further purified by size exclusion chromatography on Superose 6 10/300 Increase column in buffer containing 20 mM Hepes-Na, pH = 7.5, 100 mM NaCl, 0.005% LMNG, 0.001% CHS, and 5 mM $MgCl_2$. The peak faction of the complex was collected and concentrated to around 5.0 mg/mL for cryo-EM sample preparation.

Cryo-EM sample preparation, data collection, and processing of the CXCR3-CXCL10-DNGi-scFv16 complex

The CXCR3-CXCL10-DNG$_i$-scFv16 complex was vitrified using glow-discharged 300-mesh alloy grids (CryoMatrix M024-Au300-R12/13, Zhenjiang Lehua Technology). 6,190 movie stacks with 50 frames per movie were collected using SerialEM[2] software in the counted-Nanoprobe mode on a 300 kV Titan Krios Gi3 electron microscope. The pixel size is 0.85 Å, and the defocus ranges from -1.2 μm to -1.8 μm. Each movie stack was recorded for 2.0 s with 0.04 s exposure per frame and a total dose of 53.26 $e^-/Å^2$.

Data processes were accomplished in RELION and CryoSPARC[3,4]. After beam-induced motion correction by MotionCor2, the contrast transfer function parameters were estimated by Gctf[6]. 7,774,488 particles were auto-picked using the Laplacian-of-Gaussian method and extracted in a pixel size of 1.70 Å in RELION. Then the particles were imported into CryoSPARC, and 2,800,769 particles were selected after two rounds of 2D classification. After 2 rounds of initial model generation and hetero-refinement in CryoSPARC, 673,702 particles were retained. The particles were re-extracted in a pixel size of 0.85 Å in RELION and went through one round of 3D classification without alignment in RELION. 110,236 particles were selected for final 3D refinement, CTF refinement, and particle polishing, yielding a density map of 3.2 Å resolution.

Atomic model building and refinement

For the CXCR3$^{\kappa OR}$-AMG487-Nb6 complex, the model of CXCR3 predicted by AlphaFold was docked into the density map in ChimeraX[7]. The ICL3 of CXCR3 (residue 241-254) was replaced with the ICL3 from the kappa-opioid receptor (residue 252-273) in Coot to get chimeric CXCR3$^{\kappa OR}$. The coordinate of AMG487 generated by AceDRG was fitted into the density. The coordinates of one

cholesterol molecule (PDB: CLR), one phosphatidylcholine molecule (PDB: PCW), and one lysophosphatidylcholine molecule (PDB: LPC) were also fitted into the density map. The structure of the complex then went through iterative manual adjustment in Coot and real space refinement in Phenix[8,9]. In the final model, residues 54-338 of CXCR3 could be traced.

For the CXCR3-CXCL10-DNG$_i$-scFv16 complex, the model of CXCR3 predicted by AlphaFold, the model of CXCL10 (PDB: 1O7Y), the model of G$_i$ ternary complex and scFv16 (from PDB: 6LFO) were docked into the density map in ChimeraX. Mutations of G203A and A326S were generated in Coot[9] to get DNG$_i$. The model was refined by iterative manual adjustment and rebuilding in Coot and real space refinement in Phenix[10]. The refined model was used as a reference to rescale the amplitude of the map by LocScale[11] in the CCP-EM software package, and the scaled map was sharpened by AutoSharpen in Phenix. In the final model, residues 1-8 of CXCL10 and residues 40-336 of CXCR3 were traced.

The geometries of models were validated using MolProbity[12]. The figures of the structures were prepared in ChimeraX[7] and PyMOL (Schrödinger, LLC).

Split-luciferase-based cAMP reporter assays

HEK293T cells co-expressing CXCR3 and different mutants (in vector pcDNA3.1) along with a split-luciferase-based cAMP biosensor (GloSensor; Promega) were seeded in 96-well white clear bottom cell culture plates (Beyotime; 15,000 cells/well, 100 μL/well) in DMEM (Macgene) containing 10% FBS (Every Green). The next day, the culture medium was removed, and 40 μL/well of drug buffer (1×HBSS, 20 mM HEPES, pH 7.4) was added for 2 min at room temperature, followed by the addition of 20 μL of CXCL10 (GenScript) solutions (the initial agonist drug concentration is 10 μM, on the basis of 1/10 gradient dilution diluted) for 10 min at room temperature. To measure agonist activity for G$_{\alpha i}$-coupled receptors, 20 μL drug buffer supplemented with luciferin (25 mg/mL final concentration) and forskolin (26 μM final concentration). Luminescence intensity was quantified 5 min later. Data were analyzed using "log(agonist)" vs. "response" in GraphPad Prism 8.0. Data were normalized to the percent agonist activity of wild-type CXCR3.

Molecular dynamic simulations

The system was constructed using CHARMM-GUI[13,14] with CHARMM force fields[15,16]. The protein (or complex) was embedded in a lipid bilayer composed of 146 DOPCs and 16 cholesterols, and

consequently solvated in a water box with 0.15 M NaCl to neutralize the charge of the system. This resulted in a system of 74565 atoms in size. Prior to the MD simulation, a 5000-step restrained energy minimization was performed, followed by the 3 ns NPT equilibration with the restraints gradually relaxed. The integration time step is chosen to be 2 fs with the neighbor list updated every 20 steps. The cutoff of long-range interaction was set to 1.2 nm, with a van der Waals switch at 1.0 nm. The electrostatic interaction was calculated using Particle-Mesh Ewald summation. The temperature and pressure were controlled at 303.15 K and 1.0 bar using Nose-Hoover barostat and Parrinello-Rahman barostat. Bonds with hydrogens were constrained using LINCS algorithm. Finally, 3 copies of 1 μs production runs were conducted using Gromacs[17], and VMD[18] was employed for data analysis.

Mass spectrometry

Lipids co-purified with $CXCR3^{\kappa OR}$ were extracted and separated using a CSH C18 column (Waters). The mobile phase consisted of solvent A (60% acetonitrile aqueous solution, 0.1% formic acid, 10 mM ammonium formate) and solvent B (10% acetonitrile aqueous solution, 90% Isopropanol, 0.1% formic acid, 10 mM ammonium formate) under positive ion mode, and solvent A (60% acetonitrile aqueous solution, 10 mM ammonium formate) and solvent B (10% acetonitrile aqueous solution, 90% Isopropanol, 10 mM ammonium formate) under negative ion mode. Gradient elution conditions were set as follows: 0~2 min, 40% to 43% B; 2~2.1 min, 43% to 50% B; 2.1~7 min, 50% to 54% B; 7~7.1 min, 54% to 70% B; 7.1~13 min, 70% to 99% B; 13~13.1 min, 99% to 40% B; 13.1~15 min, 40% B. The flow rate was 0.35 mL/min.

A Q Exactive mass spectrometer (Thermo Fisher Scientific) was used to obtain MS1 and MS2 data. The MS scan method was in the range of m/z 200–2000, The MS1 resolution was 70,000, AGC was $3e^6$, and the maximum injection time was 100 ms. According to the precursor ion intensity, Top 3 ions were selected for MS2 analysis, MS2 resolution was 17,500, AGC was $1e^5$, maximum injection time was 50 ms, and collision energy (stepped an nce) were set as: 15, 30 and 45 eV. The parameters of ESI were: sheath gas of 40 L/min, aux gas of 10 L/min, spray voltage (|KV|) of 3.80 in positive ion mode and of 3.20 in negative ion mode, capillary temperature of 320°C and aux gas heater temperature of 350.

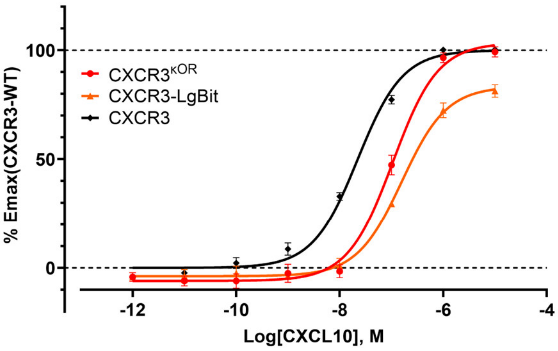

	CXCR3	CXCR3$^{\kappa OR}$	CXCR3-LgBit
EC_{50} (M)	2.23×10^{-8}	1.01×10^{-7}	1.59×10^{-7}

Supplementary Fig. S1 Validation of the activity of CXCR3$^{\kappa OR}$ and CXCR3-LgBit. cAMP responses of CXCR3, CXCR3$^{\kappa OR}$, and CXCR3-LgBit to CXCL10. cAMP responses are normalized to the percent agonist activity of wild-type CXCR3. Data are shown as mean ± s.e.m (n = 6).

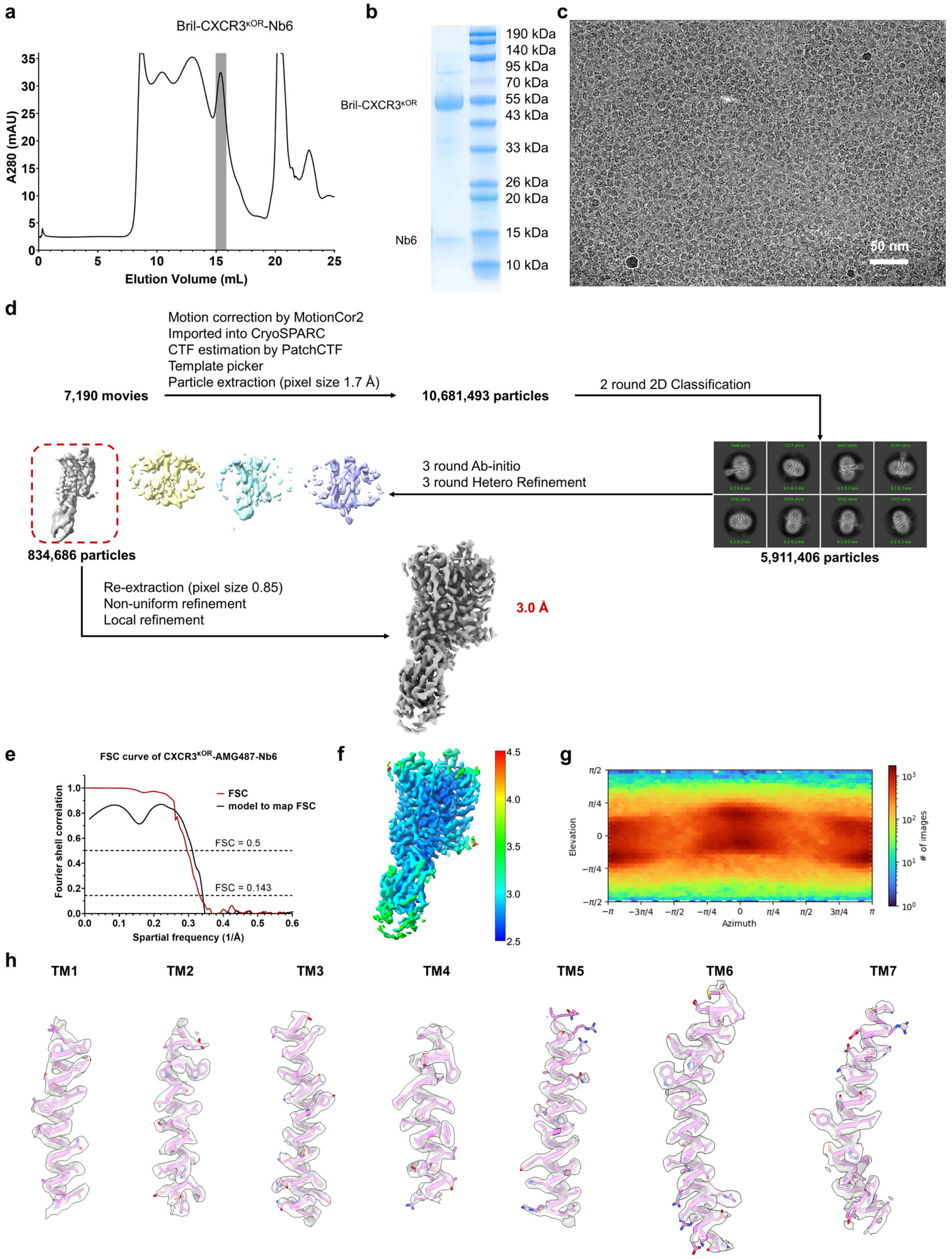

Supplementary Fig. S2 Cryo-EM analysis of the CXCR3$^{\kappa OR}$-AMG487-Nb6 complex. a The elution curve of the CXCR3$^{\kappa OR}$-AMG487-Nb6 complex on a Superose 6 Increase column. **b** SDS-PAGE of the purified CXCR3$^{\kappa OR}$-AMG487-Nb6 complex. **c** The cryo-EM image of the CXCR3$^{\kappa OR}$-

AMG487-Nb6 complex. **d** Data processing of the cryo-EM dataset. **e** The Fourier shell correlation curve of the density map. **f** Local resolution of the density map. **g** Orientation distribution of the particles used in the final refinement. **h** The electron densities of the transmembrane helices of the receptor.

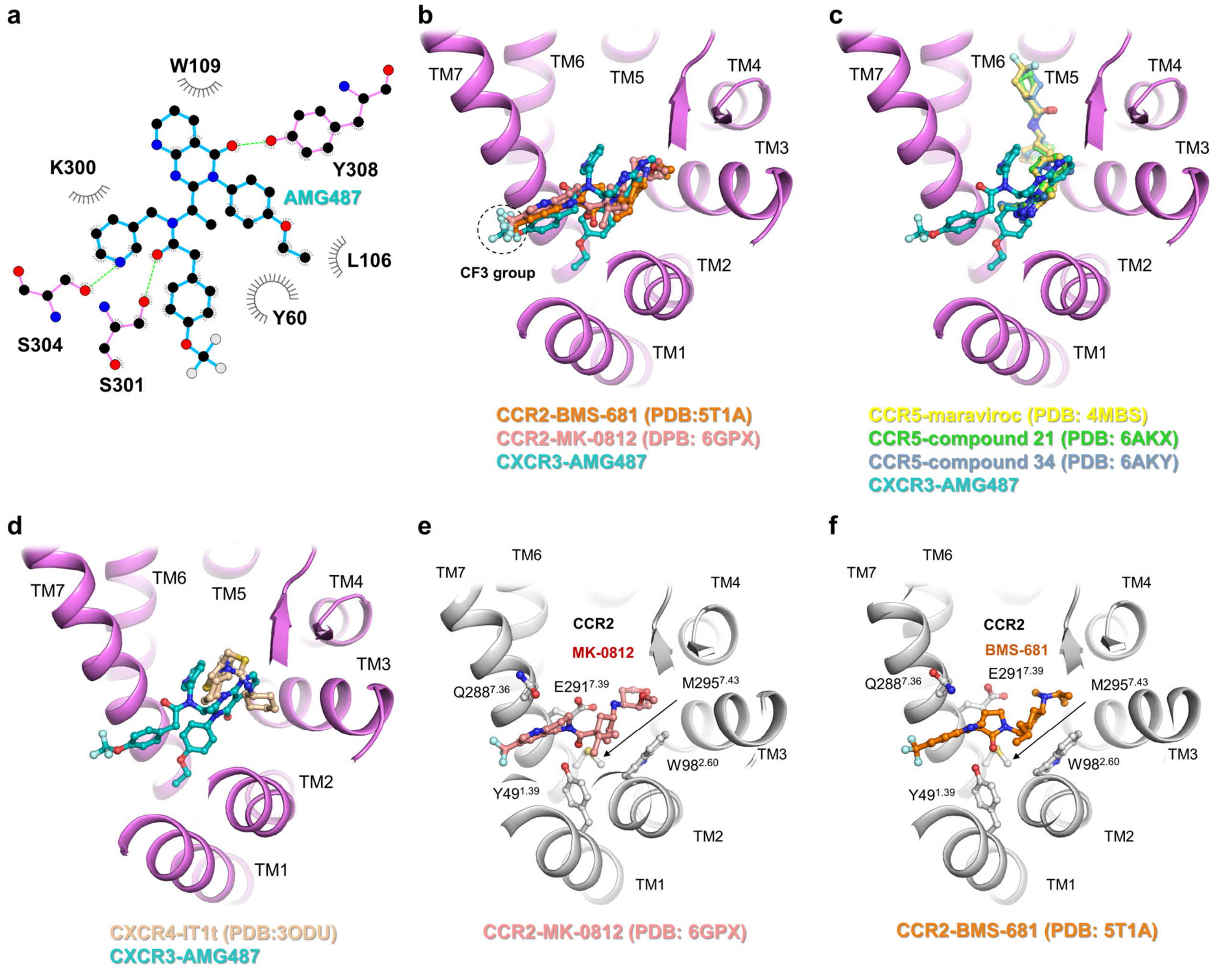

Supplementary Fig. S3 Comparation of the binding pattern of AMG487 with that of MK-0812 and BMS-681. **a** Interactions between AMG487 and CXCR3. **b** Comparison of the binding sites of AMG487 (cyan), MK-0812 (salmon), and MBS-681 (orange). The CF3 groups are indicated by a black circle. **c** Comparison of the binding sites of AMG487 (cyan), maraviroc (yellow), compound 21 (green), and compound 34 (blue). **d** Comparison of the binding sites of AMG487 (cyan) and IT1t (wheat). In b-d, CXCR3 is shown as cartoon and colored violet. Small molecule antagonists are shown as sticks. **e** The binding site of MK-0812 in the structure of CCR2. **f** The binding site of BMS-681 in the structure of CCR2. In e and f, the structure of CCR2 is shown as cartoon and colored gray, MK-0812 and BMS-681 are shown as sticks and colored salmon and orange, respectively.

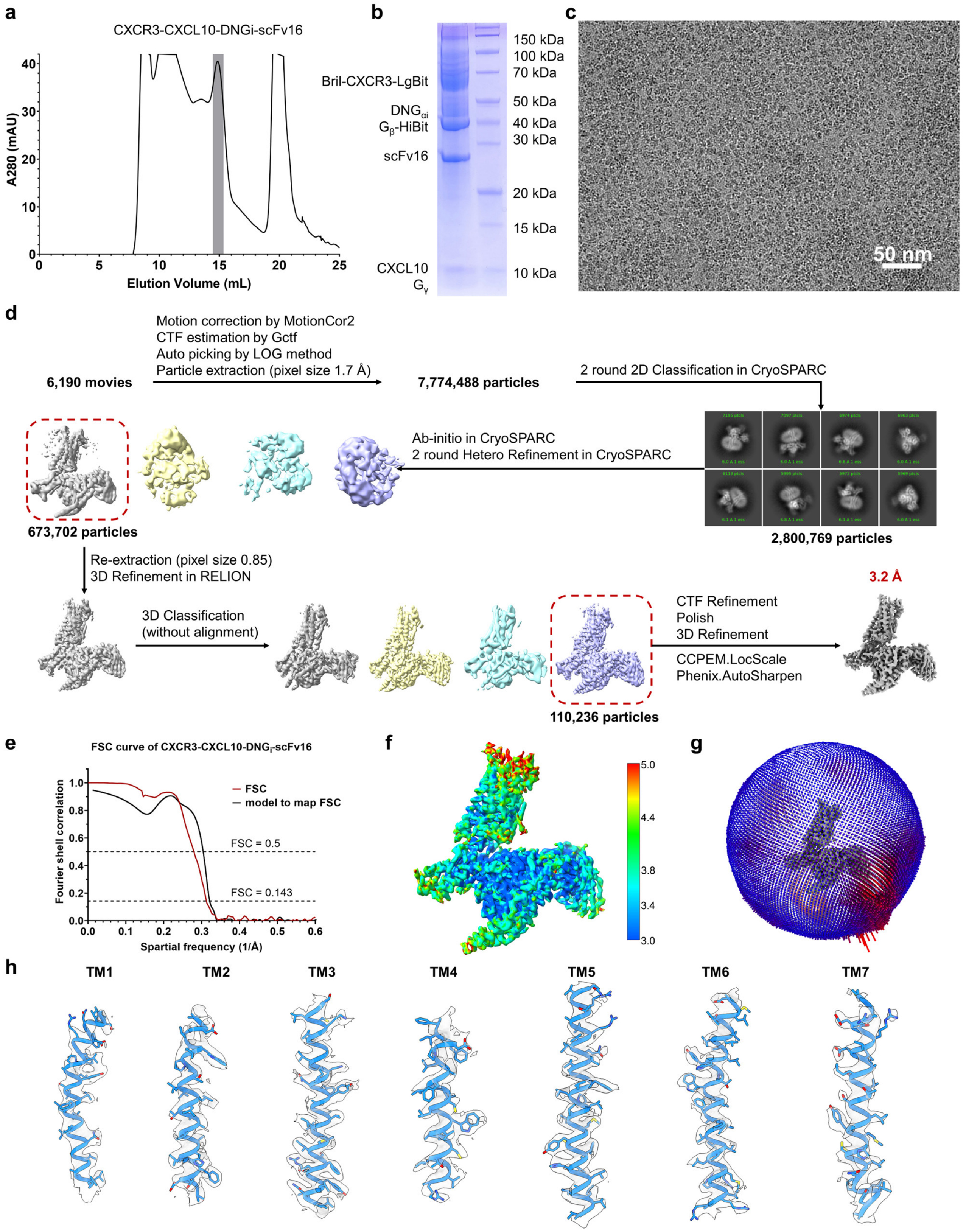

Supplementary Fig. S4 Cryo-EM analysis of the CXCR3-CXCL10-DNG$_i$-scFv16 complex. a The elution curve of the CXCR3-CXCL10-DNG$_i$-scFv16 complex on a Superose 6 Increase column. **b** SDS-PAGE of the purified CXCR3-CXCL10-DNG$_i$-scFv16 complex. **c** The cryo-EM image of the CXCR3-CXCL10-DNG$_i$-scFv16 complex. **d** Data processing of the cryo-EM dataset. **e** The fourier

shell correlation curve of the density map. **f** Local resolution of the density map. **g** Orientation distribution of the particles used in the final refinement. **h** The electron densities of the transmembrane helices of the receptor.

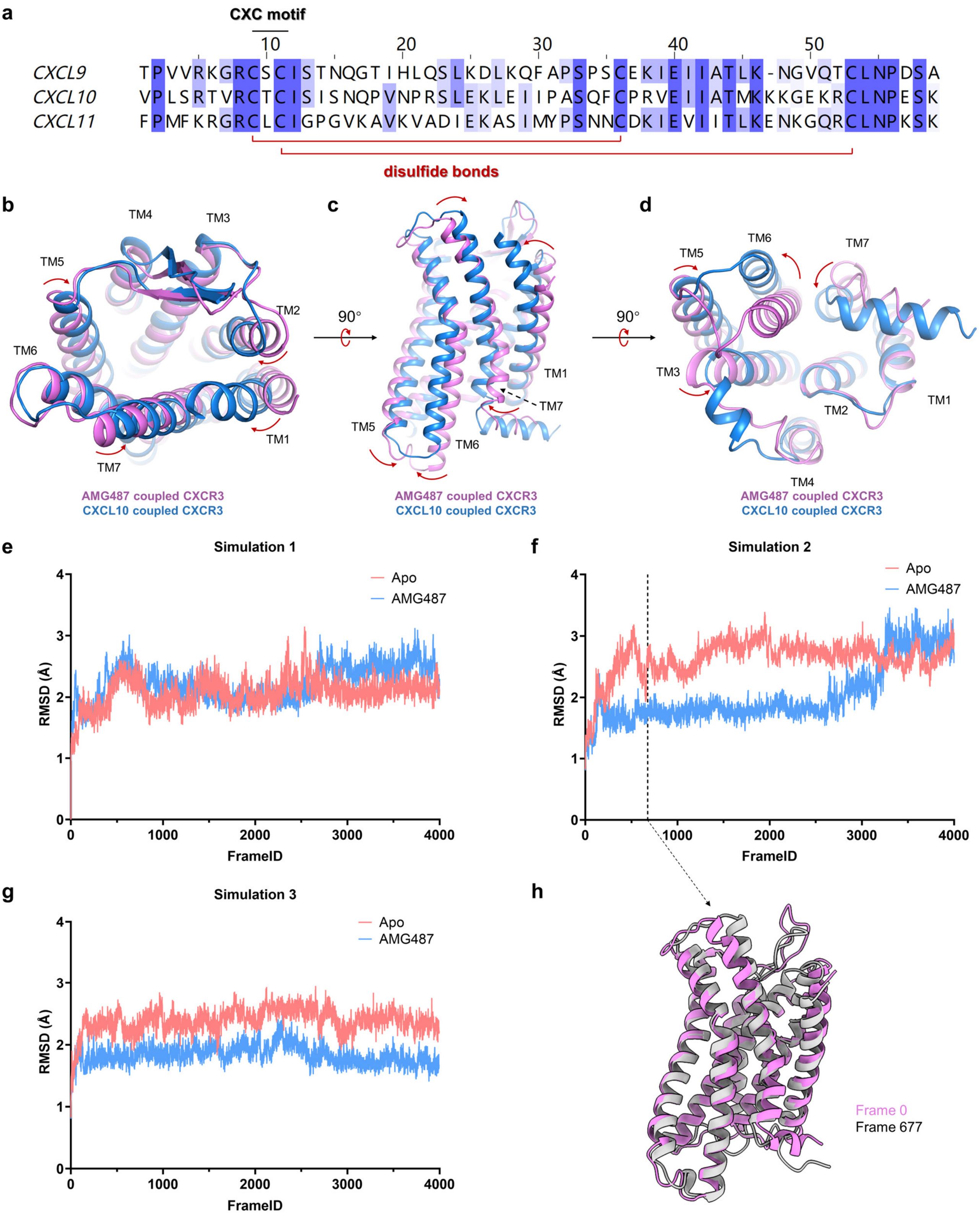

Supplementary Fig. S5 Conformation changes observed in the activation of CXCR3. a Sequence alignment of CXCL9, CXCL10, and CXCL11. Sequence alignment was accomplished in JalView and colored according to percentage identity. **b-d** Superposition of AMG-487 coupled CXCR3 (violet), and CXCL10 coupled CXCR3 (blue) in extracellular (b), side (c), and intracellular (d) view. The receptor is shown as cartoon, and displacement of the helices is indicated by red arrows. **e-f**

RMSD of CXCR3 backbone measured in three independent 1 μs MD simulations (Simulation 1 to 3). **h** Snapshots of frame 0 (violet) and frame 667 (gray) in Simulation 2 for *apo* CXCR3.

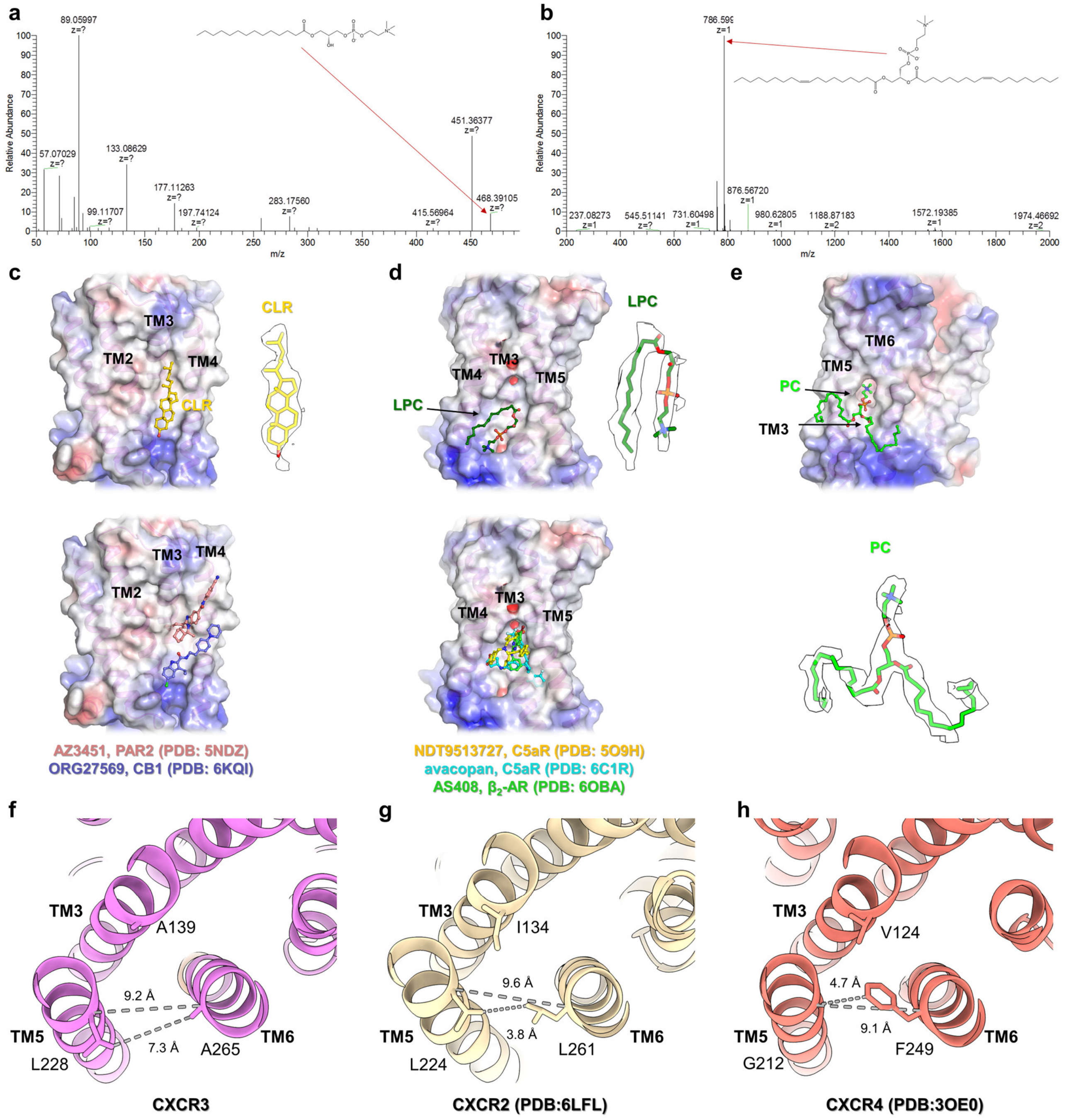

Supplementary Fig. S6 Allosteric sites in CXCR3 occupied by lipids. a Mass spectrum of co-purified lyso-phosphatidylcholine. **b** Mass spectrum of co-purified phosphatidylcholine. **c** The allosteric site between TM2-3-4 is occupied by a cholesterol molecule. AZ3451 and ORG27569, which bound in a similar site, are presented. **d** The allosteric site between TM3-4-5 is occupied by an LPC molecule. NDT9513727, avacopan, and AS408 that bind in a similar site are presented. **e** The allosteric site between TM5-3-6 is occupied by a PC molecule. In c-e, the cartoon model (colored violet) and the surface model (colored by electron potential) of CXCR3 are presented. The cholesterol, PC, and LPC molecules are shown as sticks and colored yellow, green, and dark green, respectively. The electron density of cholesterol, PC, and LPC molecules are shown aside. **f-h** Residues around the

binding pocket of phosphatidylcholine in CXCR3 compared to CXCR2 and CXCR4. CXCR3, CXCR2, and CXCR4 are shown as cartoon and colored violet, wheat, and salmon, respectively. Key residues around the pocket are shown as sticks. The distances between the α-carbon atoms and the side chains of 5.51 and 6.45 are indicated by gray dashed lines and the distances are labelled nearby.

Supplementary Table S1 Statics of data collection, data process, model refinement, and validation.

	CXCR3$^{\kappa OR}$-AMG487-Nb6 (EMDB: 36841) (PDB: 8K2W)	CXCR3-CXCL10-DNG$_i$-scFv16 (EMDB: 36842) (PDB: 8K2X)
**Data and processing**		
Magnification	105,000	105,000
Voltage (kV)	300	300
Electron exposure (e^-/ Å^2)		
Defocus range (μm)	-1.5 ~ -2.0	-1.2 ~ -1.8
Pixel size (Å)	0.425	0.85
Symmetry imposed	C1	C1
Initial particle images (no.)	10,681,493	7,774,448
Final particle images (no.)	834,686	110,236
Map resolution (Å)	3.0	3.2
FSC threshold	0.143	0.143
Map resolution range	2.5 - 4.5	3.0 - 5.0
**Refinement**		
Initial model used (PDB code)	6LFL	6LFO
Map sharpening B factor (Å^2)	202.5	77.4
Model composition		
Non-hydrogen atoms	3143	8901
Protein residues	386	1138
Ligands	4	1
B factor (Å^2)		
Protein	63.81	65.98
Ligand	52.02	78.33
R.m.s. deviations		
Bond lengths (Å)	0.003	0.004
Bond angles (°)	0.585	0.960
Validation		
MolProbity score	1.62	1.80
Clash score	5.99	9.16
Rotamers outliers (%)	0.00	0.00
Ramachandra plot		
Favored	95.77	95.45
Allowed	4.23	4.55
Outliers	0.00	0.00

Supplementary Table S2 EC_{50} values for CXCR3 and its mutants in the cAMP assay with CXCL10 as the agonist.

Mutant	$LogEC_{50}$ ± Std. Error	N Value	EC_{50} (nM)	Fold
WT	-7.651 ± 0.04	6	22.32	1.00
D52A	-6.689 ± 0.07	6	204.6	9.17
Y60A	-7.323 ± 0.06	6	58.68	2.63
W109A	-6.560 ± 0.12	6	275.7	12.4
F131A	-6.816 ± 0.07	6	152.9	6.85
Q204A	-6.769 ± 0.10	6	170.3	7.63
Y205A	-8.060 ± 0.07	6	8.706	0.39
Y271A	-7.894 ± 0.13	6	12.78	0.57
C290A	-6.970 ± 0.15	6	107.1	4.80
E293A	-5.099 ± 0.14	6	7970	357
K300A	-7.731 ± 0.08	6	18.59	0.83
S304L	-6.621 ± 0.04	6	239.3	10.7
Y308A	-7.056 ± 0.05	6	87.94	3.94

Supplementary References

1 Robertson, M. J. *et al.* Structure determination of inactive-state GPCRs with a universal nanobody. *Nat Struct Mol Biol*, doi:10.1038/s41594-022-00859-8 (2022).

2 Mastronarde, D. N. Automated electron microscope tomography using robust prediction of specimen movements. *J Struct Biol* **152**, 36-51, doi:10.1016/j.jsb.2005.07.007 (2005).

3 Scheres, S. H. A Bayesian view on cryo-EM structure determination. *J Mol Biol* **415**, 406-418, doi:10.1016/j.jmb.2011.11.010 (2012).

4 Punjani, A., Rubinstein, J. L., Fleet, D. J. & Brubaker, M. A. cryoSPARC: algorithms for rapid unsupervised cryo-EM structure determination. *Nat Methods* **14**, 290-296, doi:10.1038/nmeth.4169 (2017).

5 Zheng, S. Q. *et al.* MotionCor2: anisotropic correction of beam-induced motion for improved cryo-electron microscopy. *Nat Methods* **14**, 331-332, doi:10.1038/nmeth.4193 (2017).

6 Zhang, K. Gctf: Real-time CTF determination and correction. *J Struct Biol* **193**, 1-12, doi:10.1016/j.jsb.2015.11.003 (2016).

7 Pettersen, E. F. *et al.* UCSF ChimeraX: Structure visualization for researchers, educators, and developers. *Protein Sci* **30**, 70-82, doi:10.1002/pro.3943 (2021).

8 Adams, P. D. *et al.* PHENIX: a comprehensive Python-based system for macromolecular structure solution. *Acta Crystallogr D Biol Crystallogr* **66**, 213-221, doi:10.1107/S0907444909052925 (2010).

9 Emsley, P., Lohkamp, B., Scott, W. G. & Cowtan, K. Features and development of Coot. *Acta Crystallogr D Biol Crystallogr* **66**, 486-501, doi:10.1107/S0907444910007493 (2010).

10 Afonine, P. V. *et al.* Real-space refinement in PHENIX for cryo-EM and crystallography. *Acta Crystallogr D Struct Biol* **74**, 531-544, doi:10.1107/S2059798318006551 (2018).

11 Jakobi, A. J., Wilmanns, M. & Sachse, C. Model-based local density sharpening of cryo-EM maps. *Elife* **6**, doi:10.7554/eLife.27131 (2017).

12 Chen, V. B. *et al.* MolProbity: all-atom structure validation for macromolecular crystallography. *Acta Crystallogr D Biol Crystallogr* **66**, 12-21, doi:10.1107/S0907444909042073 (2010).

13 Jo, S., Kim, T. & Im, W. Automated builder and database of protein/membrane complexes for molecular dynamics simulations. *PLoS One* **2**, e880, doi:10.1371/journal.pone.0000880

(2007).

14 Lee, J. *et al.* CHARMM-GUI Input Generator for NAMD, GROMACS, AMBER, OpenMM, and CHARMM/OpenMM Simulations Using the CHARMM36 Additive Force Field. *J Chem Theory Comput* **12**, 405-413, doi:10.1021/acs.jctc.5b00935 (2016).

15 Klauda, J. B. *et al.* Update of the CHARMM all-atom additive force field for lipids: validation on six lipid types. *J Phys Chem B* **114**, 7830-7843, doi:10.1021/jp101759q (2010).

16 Huang, J. *et al.* CHARMM36m: an improved force field for folded and intrinsically disordered proteins. *Nat Methods* **14**, 71-73, doi:10.1038/nmeth.4067 (2017).

17 Páll, S. *et al.* Heterogeneous parallelization and acceleration of molecular dynamics simulations in GROMACS. *The Journal of Chemical Physics* **153**, 134110, doi:10.1063/5.0018516 (2020).

18 Humphrey, W., Dalke, A. & Schulten, K. VMD: visual molecular dynamics. *J Mol Graph* **14**, 33-38, 27-38, doi:10.1016/0263-7855(96)00018-5 (1996).
